# Risk factors and pregnancy outcomes associated with placental malaria in a prospective cohort of Papua New Guinean women

**DOI:** 10.1186/s12936-017-2077-4

**Published:** 2017-10-24

**Authors:** Elvin Lufele, Alexandra Umbers, Jaume Ordi, Maria Ome-Kaius, Regina Wangnapi, Holger Unger, Nandao Tarongka, Peter Siba, Ivo Mueller, Leanne Robinson, Stephen Rogerson

**Affiliations:** 1 Papua New Guinea Institute of Medical Research, Madang, Papua New Guinea; 2 Department of Medicine and Radiology, Peter Doherty Institute, University of Melbourne, Melbourne, VIC Australia; 3 Barcelona Centre for International Health Research (CRESIB), Barcelona, Spain; 4 Population Health and Immunity Division, Walter and Eliza Hall Institute, Melbourne, VIC Australia; 5 Institute Pasteur, Paris, France; 6 Burnet Institute, Melbourne, VIC Australia

**Keywords:** Placental malaria, Pregnancy, Birth outcomes, Intermittent preventive treatment in pregnancy, Insecticide-treated bed nets, Papua New Guinea

## Abstract

**Background:**

*Plasmodium falciparum* in pregnancy results in substantial poor health outcomes for both mother and child, particularly in young, primigravid mothers who are at greatest risk of placental malaria (PM) infection. Complications of PM include maternal anaemia, low birth weight and preterm delivery, which contribute to maternal and infant morbidity and mortality in coastal Papua New Guinea (PNG).

**Methods:**

Placental biopsies were examined from 1451 pregnant women who were enrolled in a malaria prevention study at 14–26 weeks gestation. Clinical and demographic information were collected at first antenatal clinic visits and women were followed until delivery. Placental biopsies were collected and examined for PM using histology. The presence of infected erythrocytes and/or the malaria pigment in monocytes or fibrin was used to determine the type of placental infection.

**Results:**

Of 1451 placentas examined, PM infection was detected in 269 (18.5%), of which 54 (3.7%) were acute, 55 (3.8%) chronic, and 160 (11.0%) were past infections. Risk factors for PM included residing in rural areas (adjusted odds ratio (AOR) 3.65, 95% CI 1.76–7.51; p ≤ 0.001), being primigravid (AOR 2.45, 95% CI 1.26–4.77; p = 0.008) and having symptomatic malaria during pregnancy (AOR 2.05, 95% CI 1.16–3.62; p = 0.013). After adjustment for covariates, compared to uninfected women, acute infections (AOR 1.97, 95% CI 0.98–3.95; p = 0.056) were associated with low birth weight babies, whereas chronic infections were associated with preterm delivery (AOR 3.92, 95% CI 1.64–9.38; p = 0.002) and anaemia (AOR 2.22, 95% CI 1.02–4.84; p = 0.045).

**Conclusions:**

Among pregnant PNG women receiving at least one dose of intermittent preventive treatment in pregnancy and using insecticide-treated bed nets, active PM infections were associated with adverse outcomes. Improved malaria prevention is required to optimize pregnancy outcomes.

## Background

In endemic areas, malaria in pregnancy poses a substantial risk for both mother and child. Globally, over 125 million pregnant women are at risk of malaria infection [1] and up to 200,000 infant deaths and 10,000 maternal deaths occur annually due to malaria in pregnancy [2–4].

In African settings, pregnant women are more vulnerable to malaria infection than non-pregnant women [4] and susceptibility is highest in young, primigravid mothers who have two to seven times higher risk of delivering low birth weight (LBW, < 2500 g) babies than multigravidae [2], while other associated poor outcomes include maternal anaemia and preterm delivery (PTD, < 37 weeks) [2, 4]. There are relatively limited data from Papua New Guinea (PNG), although previous studies have reported similar findings [5–7]. Both LBW and PTD complicating malaria in pregnancy are associated with increased risk of infant mortality [4] and high rates of cognitive impairment, learning disability and behavioural problems in children [2]. Maternal anaemia due to malaria is an independent risk factor for LBW and intrauterine growth restriction (IUGR) [8–10] and may also increase the risk of PTD and stillbirth [5, 11].

The presence of infected erythrocytes (IE) or malaria pigment (haemozoin) in the intervillous spaces of placenta is termed placental malaria (PM) [8, 12, 13]. The mature asexual stages of *Plasmodium falciparum* IE can sequester in the placenta [10] and avoid splenic clearance. Often pregnant women are asymptomatic, and the absence of detectable peripheral parasitaemia does not preclude placental infection [8, 14, 15] making detection and treatment challenging [16, 17]. Methods to detect *P. falciparum* in pregnancy include peripheral and placental blood microscopy, antigen detection tests, polymerase chain reaction (PCR) and placental histology [16]. Of these, placental histology [18, 19] and PCR are the most sensitive techniques [16, 17].

In previous studies, placental IE and macrophages containing malaria pigment detected on placental histology have been associated with decreased infant birth weight and maternal haemoglobin concentration [19]. In studies of sub-microscopic infections detected by PCR, these infections were associated with anaemia but less commonly with LBW [18, 20]. Women with malaria detected by microscopy were more likely to be anaemic and had lower haemoglobin concentrations than uninfected women [18, 19].

Malaria is hyperendemic with perennial malaria transmission in coastal areas of PNG, reaching levels rarely found outside of sub-Saharan Africa [21] and it contributes significantly to maternal mortality and LBW [6, 22]. In this setting, malaria has been associated with LBW due to both IUGR and PTD (which has also been associated with maternal anaemia) and these adverse outcomes are particularly common in primigravid women [5, 7, 22]. Since these earlier studies, there have been changes to malaria control programs during pregnancy. To investigate this further, the prevalence of PM, risk factors for PM, and maternal anaemia and birth outcomes associated with PM were studied in a cohort of women receiving at least one dose of intermittent preventive treatment in pregnancy (IPTp) and sleeping under insecticide-treated bed nets (ITN).

## Methods

### Study population and design

The study was conducted in Madang Province located on the north coast of PNG. Following written informed consent, pregnant women aged 16–49 years and between 14 and 26 weeks gestation (by fundal height) were recruited into a randomized clinical trial comparing three doses of IPTp with sulfadoxine/pyrimethamine (SP, 1500/75 mg) and azithromycin (AZ, 1 g twice daily for 2 days) as compared to a curative dose of SP and chloroquine (CQ) [22]. Women were excluded if they had tuberculosis, diabetes, renal failure, severe anaemia (haemoglobin < 7 g/dL), permanent disability or multiple pregnancies. The study protocol was approved by PNG Institute of Medical Research (IMR) Institutional Review Board (0815), PNG Medical Research Advisory Committee (08.01) and Melbourne Health Human Research Ethics Committee, Australia (2008.162). The clinical trial registration number is NCT01136850 [22].

### Clinical data collection

Basic clinical and demographic data were collected at first antenatal clinic (ANC) visit and haemoglobin concentration was measured by HemoCue^®^ device (HemoCue, Ångelholm, Sweden). At delivery additional clinical data were collected, birth weight was measured using a digital baby scale (Cupid 1, Charder Medical, Taiwan) shortly after delivery, and maternal haemoglobin was measured again. Anaemia was defined as maternal haemoglobin < 11.0 g/dL, and categorized as mild (Hb 9.0–10.9 g/dL), moderate (7.0–8.9 g/dL) and severe (< 7.0 g/dL). Symptomatic women with a positive malaria rapid diagnostic test (CareStart™ P.f/Pan combo, Access-Bio, USA) at ANC visits were considered to have clinical malaria and were treated with anti-malarials. PTD was defined based on obstetric ultrasound, performed in a subset of participants, using the earliest available dating scan.

### *Plasmodium* species diagnosis

Peripheral thick and thin blood slides collected at enrolment, follow-up visits and delivery were Giemsa-stained and examined for malaria parasite speciation and density quantification using established methods [23]. Those blood slides positive for malaria were not treated with anti-malarials.

### Placental biopsy collection, preparation and processing

Placental biopsies were collected from the maternal side of the placenta. Incisions extended from the maternal to the fetal side of the placenta without reaching the fetal membrane. Biopsies were fixed and transported in 10% neutral buffered formalin for processing at the University of Melbourne Histology Department. Sections were stained with Giemsa, cover slipped and returned to PNG for analyses. A subset of placental biopsies was examined in Barcelona, Spain for quality control.

### Placental histology evaluation

The histological analysis was performed primarily in Madang, PNG, where examiners were blinded to intervention group and clinical data. Each placental slide was classified for malaria infection using the presence or absence of three histological features: IE, malaria pigment in monocytes/macrophages, and malaria pigment in fibrin deposits [24, 25] (Table 1). All cases with parasites (acute and chronic infections) were termed active infections [24], and for analysis we combined both active and past infections, due to the relatively small number of the former.

**Table 1:** Classification of placental pathology [24, 25]

Infection stages	Description
Acute	Parasites, no pigment in monocytes or fibrin
Chronic	Parasites, pigment in monocytes and/or fibrin
Past	No parasites, pigment only
No	No parasites or pigment

### Data analysis

The placental histology data were merged with the existing clinical database, and correlated with clinical and demographic variables using the Pearson Chi squared test for categorical variables, and the Student’s t test for normally distributed continuous variables between two groups. Logistic and linear regression analyses were performed to identify risk factors and adverse maternal and infant outcomes associated with PM at delivery. Univariable associations were determined between clinical and demographic risk factors for PM and gestational age, anaemia, haemoglobin concentration, birth weight, LBW and PTD. Predictors of infant and maternal outcomes were identified from adjusted regression models including all exposure variables. All tests were two-tailed and the confidence level was set at 95%. All statistical analyses were performed using Stata 11 (Stata Corp, Texas, USA).

## Results

### Clinical and demographic characteristics of study cohort

**Fig. 1:**
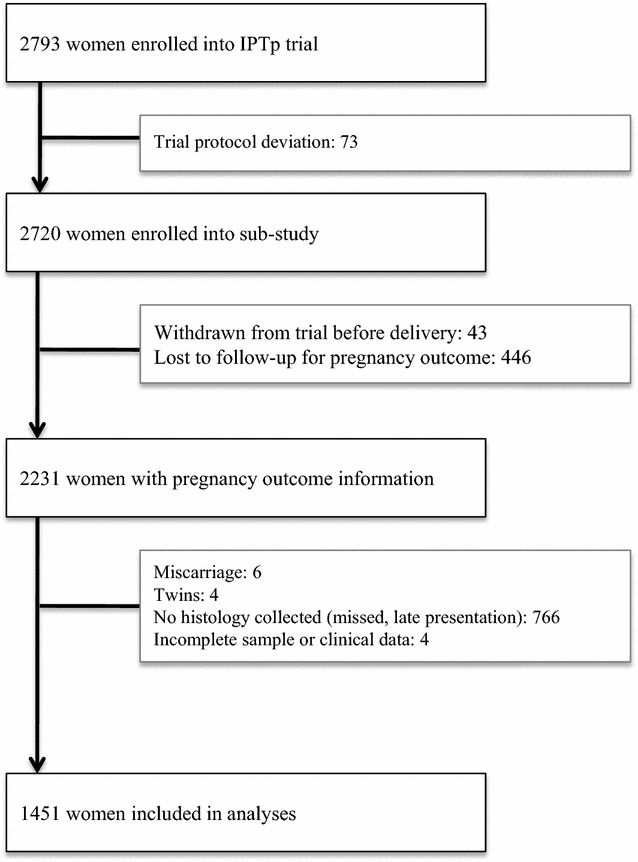
Flowchart showing the number of pregnant women enrolled for the parent study whose placental histology was collected for placental malaria investigation

A total of 1451 pregnant women with placental biopsies including their matched clinical and laboratory data were available for inclusion in this study (Fig. 1). Their demographical and clinical characteristics are summarized in Table 2. The study population included equal proportions of women from both trial arms, and 11.9% (172/1448) experienced symptomatic malaria during their pregnancy (Table 2). The mean age was 24 ± 5.4 years and mean body weight was 54 ± 9.7 kg (Table 2). Approximately half (50.9%, 738/1449) of the women were primigravid, and 26.7% (379/1420) were defined as malnourished with a mid-upper arm circumference (MUAC) < 23 cm. Most women were of coastal ethnicities and the majority resided in rural areas. They were generally subsistence farmers or earning wages and equal proportions of them used bed nets with or without insecticides (Table 2).

**Table 2:** Clinical and demographic characteristics of pregnant women with and without placental malaria

Risk factors	n	Placental malaria		p^†^
Maternal		Positive	Negative	
IPTp				
Intervention arm	726/1451	126 (46.8)	600 (50.8)	0.246
Control arm	725/1451	143 (53.2)	582 (49.2)	
Clinical malaria	172/1448	43 (25.0)	129 (75.0)	*0.020*
MUAC (cm)	379/1420	78 (20.6)	301 (79.4)	0.212
Weight (kg)	54.3 [± 9.7]	53 [± 8.0]	55 [± 10.0]	*0.007* ^♦^
Age (years)	24.5 [± 5.4]	23 [± 5.0]	25 [± 5.5]	*0.003* ^♦^
Gravidity				
Primigravidae	738/1449	164 (61.0)	574 (48.6)	*0.001*
Secundigravidae	309/1449	48 (17.8)	261 (22.2)	
Multigravidae	402/1449	57 (21.2)	345 (29.2)	
Ethnic group				
Sepik	258/1450	46 (17.1)	212 (82.2)	*0.025*
Madang/morobe	934/1450	190 (70.6)	744 (79.7)	
Highlands	125/1450	12 (4.5)	113 (90.4)	
Other	133/1450	21 (7.8)	112 (84.2)	
Place of residence				
Rural	827/1445	190 (70.6)	637 (77.0)	≤ *0.001*
Peri-urban	282/1445	41 (15.2)	241 (20.5)	
Migrant	75/1445	15 (5.6)	60 (5.1)	
Urban	261/1445	23 (8.6)	238 (20.2)	
Bednet use				
Not used	328/1445	57 (21.3)	271 (23.0)	0.064
Used, not ITN	600/1445	128 (47.8)	472 (40.1)	
Used, ITN	517/1445	83 (30.9)	434 (36.9)	
Socio-economic				
Employment				
Subsistence farmer	313/745	62 (42.2)	251 (41.9)	0.700
Self-employed	149/745	26 (17.7)	123 (20.6)	
Wage earner	283/745	59 (40.1)	224 (37.5)	
Literate	1371/1450	257 (18.8)	1114 (81.2)	0.429
Betel nut chewer	1198/1444	221 (18.5)	977 (81.5)	0.926
Smoker	282/1450	45 (16.0)	237 (84.0)	0.212
Alcohol consumer	83/1446	9 (10.8)	74 (89.2)	0.063

Data are number (%), or mean [standard deviation]

*IPTp* intermittent preventive treatment in pregnancy, *MUAC* mid-upper arm circumference, *ITN* insecticide-treated bed nets

^†^Estimated by Pearson Chi squared and ^♦ ^Student t tests, p values are shown. p < 0.05 marked in italic

### Prevalence of placental malaria infection

Placental biopsies for histological examination and delivery data were available from 1451 pregnant women. Of 1451 placentas examined, 18.5% (269/1451) showed evidence of current or past PM. There were 7.5% active infections [3.7% (54/1451) acute, and 3.8% (55/1451) chronic], and 11.0% (160/1451) past infections.

### Risk factors for placental malaria infection at delivery

In univariate analyses, women’s weight (OR 0.98, 95% CI 0.97–1.00; p = 0.014), and age (OR 0.96, 95% CI 0.93–0.98; p = 0.001), were negatively associated with PM, while first pregnancy increased the likelihood of PM (OR 1.73, 95% CI 1.24–2.40; p = 0.001) (Table 3). Risk factors for PM included living in rural (OR 3.09, 95% CI 1.95–4.88; p ≤ 0.001) or peri-urban areas (OR 1.76, 95% CI 1.02–3.02; p = 0.041), having recently migrated into the study area from other provinces of PNG (OR 2.59, 95% CI 1.27–5.26; p = 0.009), using bed nets without insecticides (OR 1.42, 95% CI 1.04–1.92; p = 0.025), and presenting with clinical malaria (OR 1.56, 95% CI 1.07–2.26; p = 0.020) (Table 3). In multivariate analyses, clinical malaria (AOR 2.05, 95% CI 1.16–3.62; p = 0.013), first pregnancy (AOR 2.45, 95% CI 1.26–4.77; p = 0.008) and rural residence (AOR 3.65, 95% CI 1.76–7.51; p ≤ 0.001) remained significant risk factors for PM (Table 3).

**Table 3:** Univariate and multivariate analyses for risk factors associated with placental malaria

Risk factors	n	Unadjusted (95% CI)	p	Adjusted (95% CI)^a^	p
Maternal					
IPTp, n = 1451					
Intervention arm	726	0.85 (0.66–1.11)	0.246	0.69 (0.47–1.03)	0.068
Control arm	725	REF	REF	REF	REF
Clinical malaria, n = 1448	172	1.56 (1.07–2.26)	*0.020*	2.05 (1.16–3.62)	*0.013*
MUAC (cm), n = 1420	379	1.21 (0.90–1.62)	0.212	1.04 (0.65–1.66)	0.881
Weight (kg)	1451	0.98 (0.97–1.00)	*0.014*	0.98 (0.96–1.00)	0.080
Age (years)	1451	0.96 (0.93–0.98)	*0.001*	1.00 (0.95–1.05)	0.979
Gravidity, n = 1449					
Primigravidae	738	1.73 (1.24–2.40)	*0.001*	2.45 (1.26–4.77)	*0.008*
Secundigravidae	309	1.11 (0.73–1.69)	0.614	1.40 (0.70–2.78)	0.344
Multigravidae	402	REF	REF	REF	REF
Ethnic group, n = 1450					
Sepik	258	1.16 (0.66–2.04)	0.612	1.06 (0.46–2.47)	0.892
Madang/morobe	934	1.36 (0.83–2.23)	0.219	0.92 (0.45–1.89)	0.825
Highlands	125	0.57 (0.27–1.21)	0.140	0.60 (1.81–2.00)	0.409
Other	133	REF	REF	REF	REF
Place of residence, n = 1445					
Rural	827	3.09 (1.95–4.88)	*≤ 0.001*	3.65 (1.76–7.51)	*≤ 0.001*
Peri-urban	282	1.76 (1.02–3.02)	*0.041*	1.32 (0.55–3.17)	0.451
Migrant	75	2.59 (1.27–5.26)	*0.009*	1.65 (0.50–5.37)	0.409
Urban	261	REF	REF	REF	REF
Bednet use, n = 1445					
Not used	328	1.10 (0.76–1.59)	0.614	1.26 (0.73–2.16)	0.402
Used, not ITN	600	1.42 (1.04–1.92)	*0.025*	1.43 (0.91–2.24)	0.123
Used, ITN	517	REF	REF	REF	REF
Socio-economic					
Employment, n = 745					
Subsistence farmer	313	0.94 (0.63–1.40)	0.753	0.64 (0.40–1.03)	0.064
Self-employed	149	0.80 (0.48–1.34)	0.399	0.83 (0.47–1.47)	0.523
Wage earner	283	REF	REF	REF	REF
Literate, n = 1450	1371	1.29 (0.69–2.42)	0.430	0.84 (0.34–2.10)	0.708
Betel nut chewer, n = 1444	1198	0.98 (0.69–1.40)	0.926	1.67 (0.89–3.13)	0.107
Smoker, n = 1450	282	0.80 (0.56–1.14)	0.212	0.96 (0.57–1.62)	0.874
Alcohol consumer, n = 1446	83	0.52 (0.26–1.05)	0.068	0.35 (0.11–1.07)	0.065

Data are odds ratios (95% confident interval) for logistic regression models, p values are shown. p < 0.05 marked in italic

*IPTp* intermittent preventive treatment in pregnancy, *MUAC* mid-upper arm circumference, *ITN* insecticide-treated bed nets, *REF* reference group

^a^Multivariate analyses included all risk factors

### Association of placental malaria with maternal anaemia, low birth weight, and preterm delivery at birth

Anaemia was highly prevalent in this population (72.6%, 961/1323). In unadjusted analyses, women with chronic (p ≤ 0.001) and past (p = 0.023) infections had lower haemoglobin concentrations than uninfected women. In adjusted analyses, only chronic infection remained associated with reduced haemoglobin concentrations (Table 4). In unadjusted analyses, women with chronic (OR 2.13, 95% CI 0.99–4.58; p = 0.053) and past (OR 1.64, 95% CI 1.06–2.53; p = 0.025) infections were at two fold increased odds of having anaemia compared to uninfected women. In adjusted analyses, only chronic infections remained associated with anaemia (AOR 2.22, 95% CI 1.02–4.84; p = 0.045) (Table 4).

**Table 4:** Association between placental malaria infection stages with birth outcomes at delivery, adjusted for confounding variables

	Hb level		Anaemia		Birth weight		LBW		GA		PTD	
PM stages	ACoeff (95% CI)^a^	p	AOR (95% CI)^a^	p	ACoeff (95% CI)^a^	p	AOR (95% CI)^a^	p	ACoeff (95% CI)^a^	p	AOR (95% CI)^a^	p
Acute	0.15 (− 0.34–0.64)	0.560	0.61 (0.33–1.10)	0.091	− 188.98 (− 323.33–54.64)	*0.006*	1.97 (0.98–3.95)	*0.056*	− 0.65 (− 5.02–3.71)	0.770	2.33 (0.86–6.35)	0.097
Chronic	− 0.87 (− 1.36–0.38)	*0.001*	2.22 (1.02–4.84)	*0.045*	− 63.64 (− 199.08–71.79)	0.357	1.22 (0.59–2.50)	0.589	− 3.39 (− 7.79–1.02)	0.132	3.92 (1.64–9.38)	*0.002*
Past	− 0.25 (− 0.56–0.05)	0.103	1.53 (0.89–2.38)	0.061	− 76.12 (− 159.07–6.84)	0.072	1.00 (0.61–1.63)	0.985	− 1.52 (− 4.27−1.23)	0.279	1.46 (0.71–3.03)	0.305
No	REF	REF	REF	REF	REF	REF	REF	REF	REF	REF	REF	REF

Data are coefficients or odds ratios (95% confident interval) for multivariate linear and logistic regression models, p values are shown. p < 0.05 marked in italic

*PM* placental malaria, *Hb* haemoglobin, *LBW* low birth weight, *GA* gestational age, *PTD* preterm delivery, *ACoeff* adjusted coefficient or *AOR* adjusted odds ratio, *REF* reference group

^a^Variables included in multivariate analyses are placental malaria infection stages, gravidity, place of residence, maternal age, clinical malaria, maternal mid-upper arm circumference and weight

The severity of anaemia was significantly associated with PM stages. Among women with chronic PM, more had moderate anaemia (35.3%, 18/51) and severe anaemia (11.8%, 6/51) compared to uninfected women (moderate anaemia 14.9%, 161/1079 and severe anaemia 3.2%, 34/1079) (p ≤ 0.001), respectively.

Low birth weight was prevalent in this study cohort (14.0%, 203/1448). In unadjusted analyses, there were significant associations between presence of acute (p = 0.005), chronic (p = 0.042) or past (p = 0.002) PM infections and reduced birth weight, while only acute PM infections (OR 1.95, 95% CI 1.00–3.80; p = 0.049) were associated with increased odds to have LBW babies. In adjusted analyses, acute PM infections were associated with reduced birth weight compared to uninfected women, and with increased risk of LBW deliveries (AOR 1.97, 95% CI 0.89–3.95; p = 0.056) compared to uninfected women (Table 4). Women with acute infections (mean birth weight, 2780 ± 692 g; p = 0.002) had birth weights on average 199 g lower than uninfected women (mean birth weight, 2979 ± 490 g).

Preterm delivery determined by ultrasound dating was observed in 8.4% (81/962) of women in this study. The timing of the ultrasound dating was compared by groups with different placental histology findings. There were no significant differences in timing of first ultrasound between groups, women with chronic PM infections were almost four times more likely to have PTD compared to uninfected women (AOR 3.92, 95% CI 1.64–9.38; p = 0.002) (Table 4). Women with acute PM infections (AOR 2.33, 95% CI 0.86–6.35; p = 0.097) also had increased odds to have PTD but the association was not statistically significant (Table 4). When duration of gestation was examined, women with chronic PM infections (mean gestational age, 271 ± 13 days) had an average gestational age approximately 4 days less than those with no PM (mean gestational age; 275 ± 13 days; p = 0.041).

## Discussion

In the context of a trial of IPTp in PNG [22], the prevalence and risk factors for PM infection are examined, and poor pregnancy outcomes associated with PM are described. In the largest survey of PM in Western Pacific region to date, risk factors for PM include residing in rural areas, being primigravid and having symptomatic malaria infection during pregnancy. The impact of PM on pregnancy outcomes varied according to the type of placental infection. Maternal anaemia and PTD were associated with chronic PM infections, whereas LBW was more common in acute PM infections.

The present study focused on *P. falciparum*-infected placentas, as this parasite was the only one detected in placentas with active infection by qPCR, and it is the major cause of adverse effects on the mother and baby world-wide in regions where malaria is endemic [1]. Previous studies in the same area showed high prevalence of *P. falciparum* malaria in pregnant women [6, 26] with PM detected in over 40% of pregnancies [7, 27]. In the present study, PM was found in 18.5% of participants, and past infections were most prevalent. As previously reported, the prevalence of acute or chronic PM was lower in women who received SP and AZ [22], suggesting this combination offered protection against active PM, although it was not associated with reductions in past infection. The lower prevalence of PM infection compared to previous studies may reflect the recent overall decline of malaria in PNG [28].

The risk factors associated with PM infections vary between different epidemiological settings where malaria is endemic. In the present study, symptomatic malaria illness during pregnancy, being in first pregnancy, and residing in rural areas were risk factors for PM in adjusted analyses. While malaria infections are often asymptomatic during pregnancy, a significant proportion of parasitaemic women have symptoms such as fever, headache, malaise and dizziness [29]; the data suggest these symptomatic infections might be a proxy for PM infection, especially in light of changing epidemiology of malaria [30].

In this cohort, primigravid women were most susceptible to PM infection, and this susceptibility decreased with subsequent pregnancy, as commonly observed elsewhere [2]. It is generally understood that primigravid women are uniquely susceptible because they lack pregnancy-specific malaria immunity in the form of antibodies and memory B cells to the pregnancy-specific parasite variant surface antigen VAR2CSA [3]. These protective antibody responses develop over subsequent pregnancies, conferring protection against PM [31].

Furthermore, the vulnerability of pregnant women to PM infection is also increased by extrinsic factors during pregnancy. Women in the present cohort who were living in rural areas had a higher risk of PM infection. The spatial variation in PM infection risk amongst the women residing at various localities may be due to the heterogeneous transmission of malaria in this area, and previous studies have confirmed higher rates of transmission in rural settings where the main vector *Anopheles punctulatus* group of mosquitoes are predominantly found compared to urban areas [21].

Anaemia was highly prevalent in this cohort, and there were associations between anaemia or lower haemoglobin concentrations and PM. Chronic PM infection was associated with a significant reduction in maternal haemoglobin concentration, and it was also associated with increased risk of anaemia in adjusted analyses, in keeping with studies from Africa [25, 32]. Women with chronic PM were at significantly increased risk of moderate and severe anaemia compared to those with no PM infections. Although anaemia was associated with PM infections, there may be other factors that have contributed to anaemia in the present cohort, such as iron and/or folate deficiency [33], splenomegaly, red blood cell genetic traits such as alpha thalassaemia, and hookworm infection, all of which are prevalent in this setting [5, 7, 34, 35].

This study is consistent with previous findings [36] demonstrating *P. falciparum* acute PM infections were significantly associated with reduced mean birth weight, which may predispose to greater risk of morbidity and mortality in infancy [12, 25]. Although previous studies from this setting had reported that chronic PM infection was associated with LBW [7, 37], acute PM infections were particularly associated with LBW in the current study. This difference may be due to different study design. In the current study, women received ITNs and between one and three courses of IPTp, whereas previous studies were observational in nature, chloroquine prophylaxis rather than IPTp was recommended but not supervised and women were not provided with ITNs, potentially predisposing to longer duration chronic infections. In keeping with this, in an observational study African pregnant women, chronic PM infection especially with massive chronic intervillositis (the accumulation of large number of white blood cells in the maternal intervillous space of placenta) was associated with LBW while acute PM infection with high parasitaemia was associated with PTD [32]. Other factors may be contributing significantly to reduced fetal growth. Most women in the present study were anaemic and the occurrence of anaemia may have had a direct effect on fetal growth [35]. Additionally, less than 30% of the cohort was malnourished, suggesting that these women could have poor nutritional status. A previous study in the same setting reported associations between poor maternal nutritional status and reduced infants’ birth weight [5].

In this cohort, chronic PM infection was associated with increased risk of PTD. PM infection may contribute to LBW via multiple mechanisms including IUGR or PTD [38]. Although the causes of IUGR and PTD differ, this study supports the notion that PM infection may be an underlying cause of both in this population, alongside other factors. In this study, 24.1 and 28.4% of all LBW and PTD, respectively, were associated with PM. A previous study in this setting had reported that PM infection was associated with LBW mostly caused by IUGR rather than PTD; however, the latter was associated with maternal anaemia [5, 22].

There are some potential limitations to the present study. First, the pregnant women enrolled in the study might not be a true representation of population in the region since most women attended ANC at advanced gestational age and were ineligible to join the study [22]. Second, the health facilities used for the recruitment of study participants were in close proximity to the urban centre, therefore the study may not have measured the full extent of malaria in distant rural settings. Third, due to sampling and technical issues placental tissues from over 500 women who completed the clinical study were not able to be assessed. Although both *P. falciparum* and *Plasmodium vivax* are endemic in PNG [6], and *P. vivax* PM infection had been reported [39], the study might have underestimated the possibility that *P. vivax* can sequester in placenta even though the mechanism is less well understood [11]. Despite this, the study remains the largest to assess for the prevalence of PM, geographic and clinical risk factors including adverse pregnancy outcomes associated with PM in the Western Pacific region.

## Conclusions

The prevalence of PM infection in Madang has declined in the last decade, probably reflecting changes in malaria transmission reported across the country. Women with symptomatic malaria during pregnancy, primigravidae, and women who lived in rural settings were at increased risk for PM, despite receiving one dose of malaria prophylaxis and sleeping under insecticide-treated bed nets. Chronic PM infections were associated with anaemia, which was highly prevalent in the study cohort; acute PM infections were associated with a doubling of the likelihood of LBW; and chronic PM infections were associated with substantially increased likelihood of PTD. Associations between different categories of PM and adverse outcomes indicate that there is still need for health policy makers to improve the access and uptake of available preventive measures to prevent malaria in pregnancy in PNG.

## Abbreviations

LBW: low birth weight
PTD: preterm delivery
PNG: Papua New Guinea
IE: infected erythrocytes
PM: placental malaria
PCR: polymerase chain reaction
IUGR: intrauterine growth restriction
IPTp: intermittent preventive treatment in pregnancy
ITN: insecticide-treated bed nets
SP: sulfadoxine/pyrimethamine
AZ: azithromycin
CQ: chloroquine
IMR: Institute of Medical Research
ANC: antenatal clinic
MUAC: mid-upper arm circumference
